# Dosimetric effects of the kV based image‐guided radiation therapy of prone breast external beam radiation: Towards the optimized imaging frequency

**DOI:** 10.1002/acm2.12511

**Published:** 2018-12-14

**Authors:** Huijun Xu, Sung‐Woo Lee, Mariana Guerrero, Byong Yong Yi, Shifeng Chen, Stewart J. Becker, Heeteak Chung, Sally B. Cheston

**Affiliations:** ^1^ Department of Radiation Oncology University of Maryland School of Medicine Baltimore MD 21201 USA; ^2^ Baylor Scott & White Health Temple TX 76508 USA

**Keywords:** IGRT, imaging frequency, kV, prone breast, tangential field

## Abstract

**Purpose:**

For prone breast treatment, daily image‐guided radiation therapy (IGRT) allows couch shifting to correct breast position relative to the treatment field. This work investigates the dosimetric effect of reducing kV imaging frequencies and the feasibility of optimizing the frequency using patient anatomy or their first 3‐day shifts.

**Method:**

Thirty‐seven prone breast patients who had been treated with skin marker alignment followed by daily kV were retrospectively analyzed. Three IGRT schemes (daily‐kV, weekly‐kV, no‐kV) were simulated, assuming that fractions with kV imaging deliver a dose distribution equivalent to that in computed tomography (CT) planning, whereas other fractions yield a dose distribution as recreated by shifting the CT plan isocenter back to its position before the couch shift was applied. Treatment dose to targets (breast and lumpectomy cavity [LPC]) and organs at risks (OAR)s (heart, ipsilateral lung) in different schemes were calculated. Patient anatomy information on CT plans and first 3‐day couch shift data were analyzed to investigate whether these factors could guide imaging scheme optimization.

**Results:**

When kV imaging frequency was reduced, the percentage dose changes (*δ*D) for breast and LPC objectives (average <1%) were smaller than those for heart and lung (average 28%–31% for D_mean_). In general, the *δ*D of no‐kV imaging was approximately that of weekly kV imaging × a factor of 1.2–1.4. Although most dose objectives were not affected, the potential higher heart dose may be of concern. No strong correlation was found between *δ*D for different kV frequencies and patient anatomy size/distance or the first 3‐day couch shift data.

**Conclusions:**

Despite resulting in lower imaging dose, time, cost, and similar target coverage, a reduction in kV imaging frequency may introduce higher heart complication risk. Daily kVs are needed more in left‐sided breast patients. A less frequent imaging schedule, if considered, cannot be individually optimized using CT anatomic features or early shift data.

## 1 INTRODUCTION

Breast cancer is one of the most common cancers in women. In 2018, approximately 266 000 women are expected to be diagnosed with breast cancer in the United States.^1^ More than 60% will have localized disease and will be considered for breast‐conserving surgery followed by radiation therapy. External‐beam radiation therapy (EBRT) continues to be used in a majority of breast cancer patients.^2^, ^3^ Excellent local control rates have been achieved by using two opposing tangential beams.^4^, ^5^ In our institution, we are increasingly utilizing methods to decrease dose to normal structures relative to target structures. Specifically, we are preferentially using prone positioning to decrease dose to the lung and heart.

Prone positioning for whole‐breast radiation has several dosimetric advantages compared to supine positioning. Particularly for patients with large pendulous breasts or large breast separations, supine positioning results in considerable dose inhomogeneity with hot spots,^6^, ^7^ increased skin toxicities, and potentially increased fibrosis^8^ with diminished cosmetic outcomes.^9^, ^10^ In the prone position, the breast falls away from the chest wall and elongates, moving the target away from organs at risk (OARs; lung and heart), as well as decreasing breast separation and improving dose homogeneity. In addition, respiratory motion of the chest wall, as well as clip motion, is markedly reduced in the prone position,^11^, ^12^ thereby decreasing intrafractional variations.

Despite these dosimetric advantages, patient position reproducibility in prone treatment is more challenging. For example, when the isocenter is in the middle of the breast instead of the chest wall,^13^ the result can be greater setup and interfractional variations.^11^ Based on the studies of setup accuracy with different image assessment methods^14^ and of cardiac sparing in left‐sided breast cancer,^15^, ^16^ daily image guidance is usually required.

The standard online setup correction for prone breast uses daily megavoltage (MV) electronic portal imaging (EPI) of the tangential beams to verify the breast position relative to the treatment fields.^17^, ^18^, ^19^ To identify day‐to‐day variation in patient positioning, the positions of the breast, ribs, and lung in the two‐dimensional (2D) MV portal images are compared with a digitally reconstructed radiograph (DRR) generated from planning computed tomography (CT) images. In some clinics, imaging with other modalities is performed as an alternative to 2D MV portal images. One example is to use kV x‐ray imaging at tangential beam angles to compare with DRR and projected treatment field edges. This not only serves a similar function as EPIs but also provides better image contrast and lower imaging dose.^20^ Another example is cone‐beam CT (CBCT), which is integrated into routine treatment to provide three‐dimensional (3D) instead of 2D anatomic information. However, routine use of CBCT for daily breast setup may be limited due to high imaging dose and potential high risk to normal tissue toxicity.^21^ Specifically, organ doses from image guidance can be increased by a factor of ten using 3D kV‐CBCT compared to 2D kV imaging.^22^ Other concerns such as higher risk of collision of patient and linear accelerator can also be an issue. Jozesf et al.^23^ determined through the use of daily CBCT that their planning target volume margins were sufficient to account for setup errors. They did not, however, investigate the treatment dosimetric impact on OARs or the whole breast.

Our institution utilizes a daily two‐step setup process for prone positioning in order to minimize interfractional variations and assure accuracy. First, at the lateral gantry angle, the laser and predefined light field are aligned with patient skin marks and set to the proper source‐to‐surface distance. From this point, the gantry is rotated so that the kV source is at the actual treatment angle. The kV‐imaging‐based alignment is performed, and couch shift correction is made in the vertical and longitudinal directions to match the breast contour, clips, and chest wall with the DRR.

Although daily kV imaging assures accuracy, it increases the imaging radiation dose to the patient, patient on‐couch time, and the use of healthcare resources. We therefore investigated whether a less frequent imaging schedule could be utilized for prone breast EBRT while maintaining acceptable doses to targets and OARs. We specifically addressed this question by comparing three different kV imaging interval schemes in regard to treatment doses to target structures and OARs. We also considered patient CT anatomy information and early kV‐based couch correction data to determine whether either of these factors could help to direct optimal kV imaging frequency for individual patients.

## 2 MATERIALS AND METHODS

### 2.A Patients and planning

Anonymized data from 37 patients undergoing postoperative EBRT for breast cancer were retrospectively included for this IRT‐approved study. Of these, 19 had left‐sided disease and 18 had right‐sided disease. All had T1/T2, N0, and infiltrating ductal carcinoma. Breast volumes ranged from 246 to 2673 cc. The prescribed dose (4256 cGy in 16 fractions) to the breast was delivered by two tangential fields with control points or dynamic wedge. Most patients also received a boost of 1000 cGy in four fractions in the supine position. Data on the boost plan were not included in this study.

Target and OAR structures were outlined by one physician to avoid inter‐observer contouring differences. Targets included the whole breast and lumpectomy cavity (LPC), and OARs included the ipsilateral lung and heart. (Note that the heart was contoured only for patients with left‐sided disease.) Our departmental guidelines mandate that the mean percentage of heart receiving more than 30 Gy (V_30_) be <5%, with a mean dose <3 Gy. However, our goal is to minimize heart dose as much as possible, based on the work of Darby et al.,^24^ in which the rate of cardiac complications increased with dose with no starting threshold. Therefore, a heart mean dose <1 Gy was preferred. Our dosimetric goals were to minimize the volume of breast tissue receiving >107% and achieve V_105%_ < 75 cc per American Society for Radiation Oncology guidelines.^25^

All patients were set up with skin markers for daily kV imaging treatment to facilitate accurate targeting to minimize dose deviation per our institution's guidelines. A prone Qfix breast board (QFix; Avondale, PA) was used to allow both arms to be elevated on hand pegs above the patient's head, which was turned away from treatment site. Patient positioning alignment was then performed in two steps: (a) skin marker alignment to laser and/or a 10 × 10‐cm^2^ light field at collimator 45° or 135° and gantry 90° or 270° on the side of the treatment breast; followed by (b) kV imaging and couch shifting for further patient position correction.

### 2.B kV imaging and image registration

A free‐breathing noncontrast fan‐beam CT scan was acquired with 3‐mm slice thickness on a Philips Big Bore CT scanner (Philips Healthcare; Cleveland, OH), and treatment planning was performed using RayStation V.6 (Raysearch Laboratory; Stockholm, Sweden). A DRR was generated for registration with kV 2D imaging. kV imaging at the actual treatment tangential beam angle was acquired by the Varian Clinac series (iX and Trilogy; Varian Medical Systems; Palo Alto, CA) four‐dimensional (4D) Integrated treatment Console system for each patient. The kV image protocol for breast, with acquisition parameters of 73–95 kV, 200 mA, and 25–200 ms, was used. kV alignment against DRR and couch shift were performed each time by a therapist based on matching breast (body) contour and chest wall. A total of 16 kV images and associated couch shifts for each patient were collected from the offline review module in Varian ARIA (Varian Medical Systems) for image‐guided radiation therapy (IGRT) scheme simulation and data analysis.

### 2.C Three kV IGRT schemes: simulation and analysis

Daily couch shift data for kV IGRT after skin marker alignment were used to simulate three kV IGRT schemes: (a) daily kV; (b) weekly kV; and (c) no kV. In the daily kV scheme, kV alignment was used for every fraction, as our current practice dictates; for weekly kV, kV imaging was used only on the first day of the week; in the no kV scheme, no kV was used for any fraction. It is assumed that each fraction with kV imaging delivers the same dose distribution as the CT‐based plan, whereas fractions with no kV yield a dose distribution as recreated on the CT plan by shifting the isocenter back to where it was before the couch shift was applied. In other words, we assumed that daily KV treatment can be represented by the original CT plan (for 16 fractions in total), that weekly kV treatment has the accumulated dose distribution of four fractions of the original CT plan and 12 fractions of new plans with a shifted isocenter, and that no kV treatment results in accumulated dose of 16 fractions of new plans with shifted isocenter.

All treatment planning and dose recomputation/accumulation for the plans involved with shifted isocenter were performed in RayStation. All dose data for targets and OARs were then collected to be analyzed. The percentage dose changes [*δ*D(%)] for each structure between daily kV and weekly kV and between daily kV and no kV were denoted as *δ*Dw‐d(%) and *δ*Dn‐d(%), respectively. For this analysis:

$$\begin{array}{c} \delta \mathrm{Dw}\;-\;d=(\mathrm{Dw}\;-\;\mathrm{Dd})/\mathrm{Dd}\times 100\%,\;\mathrm{and}\;\delta \mathrm{Dn}\;-\;d=(\mathrm{Dn}\;-\;\mathrm{Dd})/\mathrm{Dd}\times 100\%, \end{array}$$

where Dd, Dw, and Dn represent structure dose as a result of using the daily kV, weekly kV, and no kV schemes, respectively. Correlations for OAR *δ*Dw‐d and *δ*Dn‐d for all structures were calculated.

Patient anatomy information on the CT plan (including breast volume and target‐to‐OAR distances) and first 3‐day couch shift data were analyzed to investigate whether either or both could be used to reduce the need for kV imaging every day. Their relationship with dose changes resulting from different kV imaging frequency was analyzed. Patient anatomy on the CT plan is information that is obtained at the planning phase, and the first 3‐day couch shift data have potential to be considered at an early treatment phase for kV imaging frequency optimization of future treatment. In this study, patient CT anatomy information refers to breast volume, shortest distances between breast centroid and chest wall, between breast centroid and lung, between breast centroid and heart, between LPC and chest wall, between LPC centroid and lung, and between LPC centroid and heart. (The centroid of a structure was defined as the mass centroid in RayStation.) The first 3‐day couch shift data refer to the average and maximum kV shift data at the first three fractions in vertical and longitudinal directions and their total numbers.

## 3 RESULTS

The statistics of patient CT anatomy for our 37 prone breast patients were summarized in Table 1.

**Table 1:** Statistics of patient CT anatomy for 37 prone breast patients’ mean value ± standard deviation

Breast volume (cc)	BRSTc_heart (cm)	BRSTc_lung (cm)	BRSTc‐CW (cm)	LPCc‐heart (cm)	LPCc‐lung (cm)	LPCc‐CW (cm)
1099 ± 618	3.7 ± 3.1	3.2 ± 3.0	1.6 ± 2.9	5.7 ± 3.1	4.6 ± 3.1	2.8 ± 2.9

BRSTc_heart: shortest distance between breast centroid and heart; BRSTc_lung: shortest distance between breast centroid and lung; BRSTc‐CW: shortest distance between breast centroid and chest wall; LPCc‐heart: shortest distance between LPC centroid and heart; LPCc‐lung: shortest distance between LPC centroid and lung; LPCc‐CW: shortest distance between LPC and chest wall.

**Figure 1:**
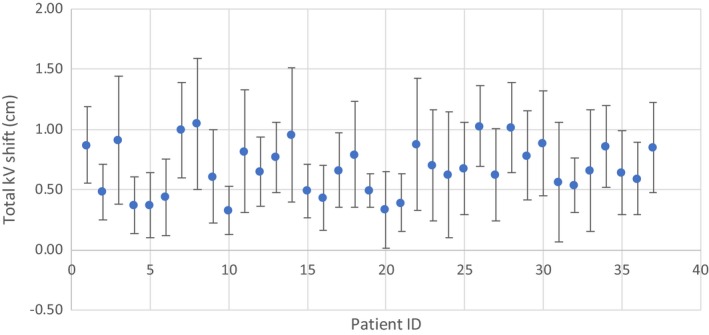
Daily KV total couch shift distribution (average ± 1 standard deviation) of 16 fractions for 37 prone patients after skin marker alignment. Patients are in the order of increasing breast volume, from 246 to 2,673 cc.

The daily kV shift data for 37 patients were used to simulate different kV imaging frequency. Figure 1 shows the average ±1 standard deviation (SD) of absolute daily kV shift magnitude after skin marker alignment is performed. The data were plotted in the order of patient breast volume. The shift magnitude ranged widely from day to day, and the average magnitude was around 3–10 mm. No trend was obvious between shift magnitude and breast volume, which means that they can be considered independent of each other.

### 3.A Dosimetric effects of reducing kV frequency

Table 2 summarizes the relative number of dose objectives that failed to be met as a result of switching from the daily kV imaging scheme to the weekly kV or no kV imaging schemes for 37 cases. For target structures, the weekly kV scheme slightly improved in one case relative to the breast V_107_ hotspot (by <1 cc); whereas the no kV scheme improved two cases for breast D_95_ (by 0.3‐1.1%) and one case for breast V_107_ (by <1 cc) but degraded another case for other breast and LPC objectives (by 1%). Overall, dose change as a result of reduced kV frequency for targets can be either slightly better or worse. As to OARs, there were no changes in dose objective failure for heart V_30_ and lung V_20_, but in two cases the heart D_mean_ exceeded 1 Gy. In general, less frequency of kV imaging does not result in any benefit to OAR dosimetry.

**Table 2:** The number of weekly‐kV and No‐kV cases that fail to meet dose objectives relative to daily‐kV benchmark for 37 patients. “+” denotes the degradation and “‐”denotes the improvement relative to daily‐kV

	Breast D_95_ < 95%Rx	Breast D_max_ < 110%	Breast V_105_ < 75 cc	Breast V_107_ < 0	LPC D_95_ < 100%Rx	Heart D_mean_ < 1 Gy	Heart V_30_ < 5%	Lung V_20_ < 15%
Weekly‐kV — Daily‐KV	0	0	0	−1	0	+2	0	0
No‐kV — Daily‐kV	−2	+1	+1	−1	+1	+2	0	0

**Figure 2:**
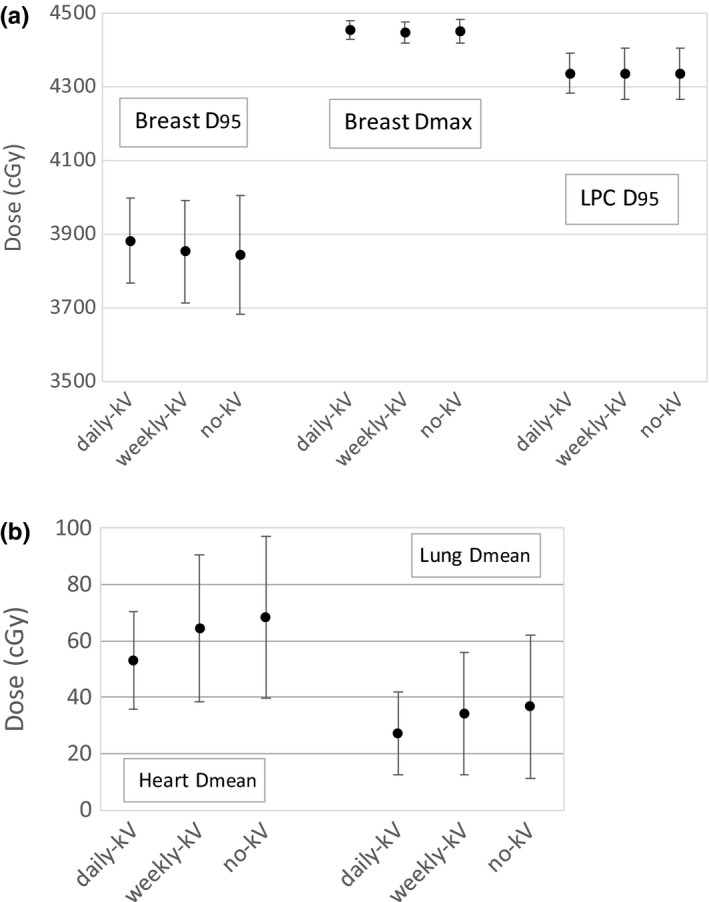
Average (avg.) ± 1 standard deviation (SD) of dose metrics for (a) target breast D_95_, D_max_, and LPC D_95_; and (b) organs at risks (OAR) heart D_mean_ and ipsilateral lung D_mean_ for daily kV, weekly kV, and no kV schemes.

Figure 2 illustrates the average distributions of Breast D_95_, Breast D_max_, LPC D_95_, Heart D_mean_, and ipsilateral lung D_mean_ as kV frequency was reduced from daily kV to weekly kV to no kV. The target dose did not seem to change with the imaging scheme. All three had <1% difference in mean, while the SD increased as imaging frequency decreased to weekly and none. Although most of the dose objectives were still acceptable after this degradation (Table 2), a potentially higher risk to the heart may be a concern. The average heart D_mean_ for the 37 patients for daily kV, weekly kV, and no kV were 53, 64, and 68 cGy, respectively, yielding a 21% *δ*Dw‐d and a 28% *δ*Dn‐d. For the worst scenario, heart D_mean_ was raised to 121 cGy (weekly kV) and 134 cGy (no kV) from 60 cGy (daily kV).

**Figure 3:**
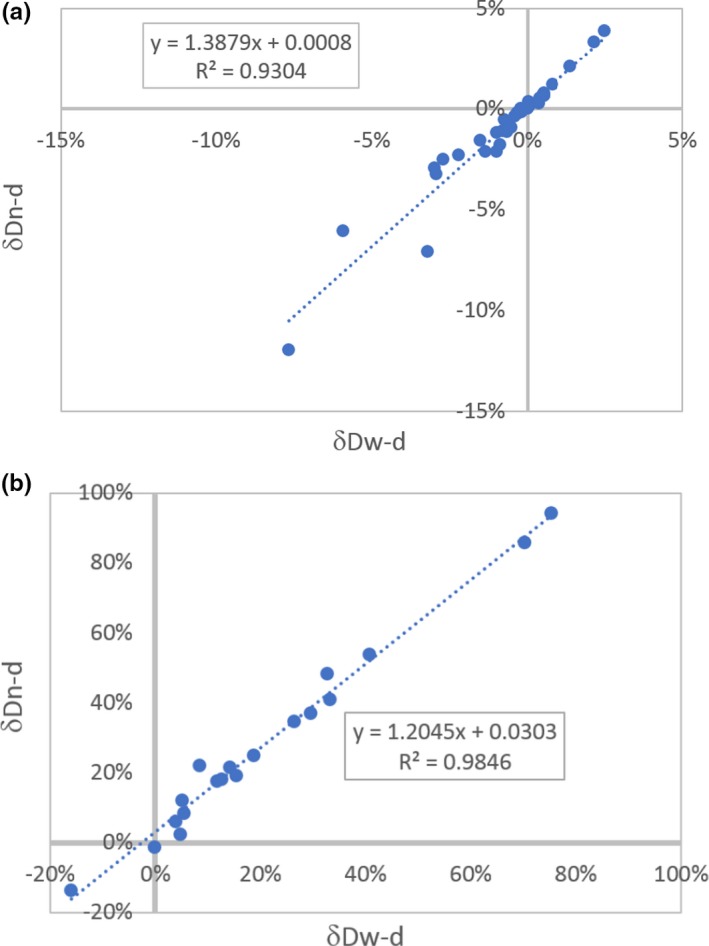
Two examples of linear relationship between percentage dose change of weekly kV and daily kV schemes (δDw‐d) and percentage dose change of no kV and daily kV schemes (δDn‐d) for (a) breast D_95_; and (b) heart D_mean_. Dots are data points and the dashed line is for linear fitting. Their correlation coefficients are R^2^ = 0.93 and 0.98, respectively, showing a strong linear relationship between δDw‐d and δDn‐d, regardless of which target or organs at risks (OAR).

Linear fittings were performed between *δ*Dw‐d and *δ*Dn‐d for targets and OARs. As Table 3 shows, the dose changes when going from daily kV to weekly kV would be magnified for dose changes going from daily kV to no kV. This linear correlation is strong and does not vary much with patients. The *R* ^2^ values of linear fitting for different structures are 0.96 on average. The slope numbers represent how much *δ*Dw‐d was magnified to approximate *δ*Dn‐d. In general, *δ*Dn‐d can be approximated by *δ*Dw‐d times a factor of 1.1 to 1.39, with an average of 1.28. The factor is dose/metric specific. Figure 3 illustrates two examples of linear relationship between *δ*Dw‐d and *δ*Dn‐d for breast D_95_ and heart D_mean_. This correlation indicates that dosimetric changes from one imaging scheme can predict dosimetric changes in another.

**Table 3:** *R* ^2^ and slope numbers and their average corresponding to linear fitting for percentage dose changes between weekly kV and no kV for targets and OARs

	Breast D_95_	Breast D_max_	LPC D_95_	Lung D_mean_	Lung D_max_	Heart D_mean_	Heart D_max_	Avg.
*R* ^2^	0.93	0.92	0.98	0.96	0.98	0.98	0.98	0.96
Slope	1.39	1.25	1.1	1.31	1.32	1.2	1.24	1.28

### 3.B Patient CT anatomy and first 3‐day couch shift data for kV frequency optimization

Because OAR percentage dose change seems more sensitive to kV frequency change than target percentage dose change, OAR dose is likely the main concern when determining whether to use reduced kV imaging frequency. To determine the possibility of predicting OAR percentage dose change for different kV imaging schemes based on patient anatomy or initial couch shifts, correlation analyzes were performed between (a) OAR *δ*Dw‐d and patient CT anatomy and (b) OAR *δ*Dw‐d and first 3‐day couch shift data. Same correlation analyzes were also performed between (a) OAR *δ*Dn‐d and patient CT anatomy and (b) OAR *δ*Dn‐d and first 3‐day couch shift data.

The resulting correlation coefficients are listed in Tables 4 and 5. The average correlation coefficients with patient CT anatomy parameters for heart are −0.14(*δ*Dw‐d) and −0.15(*δ*Dw‐n) and for lung are −0.27(*δ*Dw‐d) and −0.24(*δ*Dw‐n), respectively. The average correlation coefficients with first 3‐day couch shift for heart are 0.04(*δ*Dw‐d) and 0.07(*δ*Dw‐n) and for lung are 0.08(*δ*Dw‐d) and 0.12(*δ*Dw‐n), respectively. Therefore, the dose change sensitivity to kV frequency is not necessarily related to the size of the target, target‐to‐OAR distance, or couch shift data from the early treatment phase.

**Table 4:** Correlation coefficients between percentage weekly kV dose change and percentage no kV dose change for OARs and patient CT anatomy

CT anatomy	Heart D_mean_		Heart D_max_		Lung D_mean_		Lung D_max_	
	Weekly‐KV	No‐kV	Weekly‐KV	No‐kV	Weekly‐KV	No‐kV	Weekly‐KV	No‐kV
BRST volume	0.03	0.01	−0.20	−0.21	−0.17	−0.08	−0.26	−0.25
BRSTc_heart	−0.23	−0.22	−0.33	−0.31	−0.21	−0.14	−0.23	−0.22
BRSTc_lung	0.28	0.26	0.20	0.19	−0.31	−0.27	−0.37	−0.38
BRSTc‐CW	−0.06	−0.08	−0.20	−0.20	−0.32	−0.27	−0.35	−0.35
LPCc‐heart	−0.21	−0.19	−0.36	−0.31	−0.16	−0.09	−0.13	−0.14
LPCc‐lung	−0.11	−0.14	−0.30	−0.35	−0.27	−0.22	−0.37	−0.36
LPCc‐CW	−0.10	−0.14	−0.31	−0.35	−0.29	−0.25	−0.34	−0.35

BRST: breast; CW: chest wall; BRSTc: breast centroid; LPCc: lumpectomy cavity centroid.

**Table 5:** Correlation coefficients between percentage weekly kV dose change and percentage no kV dose change for OARs and first‐3‐day shift data

First‐3‐day shift	Heart D_mean_		Heart D_max_		Lung D_mean_		Lung D_max_	
	Weekly‐KV	No‐kV	Weekly‐KV	No‐kV	Weekly‐KV	No‐kV	Weekly‐KV	No‐kV
Max VRT shift	0.04	0.13	−0.03	0.05	0.18	0.21	0.06	0.12
Max LNG shift	0.08	0.04	0.19	0.17	−0.06	−0.07	−0.10	−0.07
Max total shift	−0.04	0.00	0.01	0.06	0.11	0.13	0.01	0.06
Mean VRT shift	−0.06	0.01	−0.13	−0.08	0.35	0.39	0.22	0.29
Mean LNG shift	0.05	0.02	0.19	0.19	−0.06	−0.03	−0.08	−0.05
Mean total shift	0.10	0.13	0.14	0.18	0.23	0.27	0.14	0.20

LNG: longitudinal; VRT: vertical.

## 4 DISCUSSION

To our knowledge, this is the first study to investigate the dosimetric effect of using different kV IGRT frequencies for prone breast EBRT. Our aim was to evaluate the necessity of daily imaging and the possibility of implementing reduced imaging for patient positioning verification. In data from 37 breast cancer patients with differing breast sizes treated in the prone position, the dosimetric effects of using reduced imaging schemes were compared in terms of delivered doses to targets and OARs. Besides skin marker alignment that is used in our procedure, when the weekly kV or no kV imaging schemes were used, a small dose change was noted for breast and LPC, whereas a relatively larger dose change (in percentage) was noted for heart and lung. The results of this study also suggested that the patient's anatomy on the planning CT or couch shift data from the early treatment phase cannot be directly used for identification of optimal imaging frequency for each patient.

Lung complications, such as radiation pneumonitis and secondary cancer, are unlikely to be of concern given the low absolute dose involved in our prone breast EBRT using tangential fields. For example, Jo et al.^26^ proposed a V_5_ < 65% criterion to predict symptomatic radiation pneumonitis; Berrington de Gonzalez et al.^27^ stated that most second solid cancers in 182 057 5‐yr breast cancer survivors were not related to radiotherapy.^28^ Radiation‐induced heart disease, in contrast, is a relevant focus of increasing concern. Data from Pierce et al.^29^ suggested lowering the mean heart dose for left‐sided breast cancer by systematically monitoring the heart dose delivered. Darby et al.^24^ found that the rate of heart problems has no starting threshold and increased by 7.4% per Gy dose. Therefore, for our worst simulated case, dose increases of 52 cGy (weekly kV) and 65 cGy (no kV) relative to the daily kV scheme may increase the heart risk by 4% and 5%, respectively. On the other hand, similar dose changes were not regarded as significant by Jacob et al.^30^ Nevertheless, to minimize the heart dose, it is necessary to utilize daily kV imaging for patients with left‐sided breast cancer. For right‐sided breast cancer, in which the heart dose is usually much lower, a reduced kV frequency may be considered.

The advantages of using a reduced kV imaging scheme for prone breast EBRT include (but may not be limited to): shorter patient on‐couch time, lower imaging dose, and lower associated healthcare costs. Our data demonstrate that, on average, kV imaging time takes 1.5 min and the rest of time for setup and treatment time requiring 8 min per fraction. The weekly kV scheme and no kV scheme consequently would produce on‐couch time reductions of 24 (15%) and 32 (20%) min, respectively, for a 16‐fraction treatment. This might help those patients who have difficulties lying in the prone position. The imaging dose/cost is reduced by approximately 75% and 100% for the weekly kV and no kV imaging schemes, respectively. In combination with other studies including imaging effective dose,^31^ it is straightforward to ascertain the gain/loss of using different imaging frequencies.

Although this study analyzed only 2D kV imaging data, the findings are also useful for other imaging modalities, such as 2D MV portal imaging. This standard imaging modality for prone breast EBRT, if used, would be expected to yield shift magnitudes and directions similar to those of kV imaging data for the same patient. Therefore, similar dosimetric effects should be found for prone breast patients using different MV imaging frequencies. It is not clear whether the 3D information on breast shape and rotations of the body that is lacking on 2D imaging may affect our findings. Setup errors in the direction of the tangential beams (perpendicular to the imager) cannot be detected.^32^ However, Becker et al.^33^ stated that these changes, detectable on CBCT, are likely to have some clinical impact on intensity‐modulated radiation therapy (IMRT) but not tangent fields. Hsu et al.^34^ pointed out the feasibility of using MV in lieu of CBCT as long as an adequate margin (≥1.5 cm) is utilized. Our future work will explore patient data using CBCT for optimal imaging frequency.

## 5 CONCLUSIONS

This study demonstrates the dosimetric effect of reducing kV imaging frequency for prone breast positioning verification: a relatively small change to target structures and larger dose increases to heart and lung. Although most dose objectives are not affected, a potentially higher heart dose may be a concern in left‐sided breast cancer when using a reduced kV imaging scheme. Therefore, we decided to continue daily kV imaging for left‐sided breast cancer patients. A less frequent imaging schedule may be considered for patients with right‐sided disease to reduce on‐couch time, lower the imaging dose, and reduce cost. However, the optimal kV frequency is difficult to predict and individualize based on patient CT anatomy information or first‐three‐fraction couch shift data. More research is needed to optimize the imaging frequency for prone breast EBRT.

## CONFLICT OF INTEREST

None.
